# Supplementary material for: LUND-PROBE – LUND Prostate Radiotherapy Open Benchmarking and Evaluation dataset
Source: Sci Data. 2025 Apr 11;12:611. doi: 10.1038/s41597-025-04954-5 (PMC11992069; doi:10.1038/s41597-025-04954-5)
Supplement: Supplementary file 1 — Supplementary Information [file 41597_2025_4954_MOESM1_ESM.docx]

## Tables

Table S1. Extensive descriptive information, file name and relative location of all data included in the base part and extended part for the 432+35 patients, respectively. Clinical naming standard was propagated to used data file names. All patients had three fiducial markers inserted and all patients had all three center of mass marker positions defined.

| **Image volume or organ segmentation name** | **Data type** | **Defined in the base part of the dataset (432 patients)** | **Defined in extended part dataset (35 patients)** | **Defined in geometry** | **DL generated by commercial software or nnUNet** | **Clinically verified. (If DL generated it was potentially edited in the clinical workflow)** | **Folder location (multiple if defined in multiple geometries)** | **Corresponding file name** | **Description** |
| --- | --- | --- | --- | --- | --- | --- | --- | --- | --- |
| MRI original | NIfTI | Yes | Yes | MRI | No | Yes | /Patient/MR_StorT2 | image.nii.gz | MRI original volume acquired from MRI scanner. |
| MRI fiducial marker positions | NIfTI | Yes | Yes | MRI | No | Yes | /Patient/MR_StorT2 | mask_MRI_T2_coords_fiducials.nii.gz | Mask with 3 mm radius spheres around each fiducial marker center of mass point. |
| MRI fiducial marker positions text file | Txt | Yes | Yes | MRI | No | Yes | /Patient/MR_StorT2 | MRI_T2_DICOM_coords_fiducials.txt | Fiducial marker center of mass point in MRI DICOM coordinates. |
| sCT original | NIfTI | Yes | Yes | sCT | Yes | Yes | /Patient/sCT | image.nii.gz | sCT original volume created from original MRI volume using Spectronic MriPlanner. |
| sCT registered and resampled to MRI volume | NIfTI | Yes | Yes | MRI | No | No | /Patient/sCT | image_reg2MRI.nii.gz | sCT registered and resampled to MRI volume. sCT warning text removed. |
| Dose distribution original | NIfTI | Yes | Yes | sCT but not resampled to sCT voxel size | No | Yes | /Patient/sCT | dose_original.nii.gz | Original dose matrix with clinical voxel size |
| Dose distribution registered and resampled to sCT | NIfTI | Yes | Yes | sCT | No | No | /Patient/sCT | dose_interpolated.nii.gz | Interpolated and resampled dose distribution matrix to sCT geometry and voxel size. |
| Dose distribution registered and resampled to MRI | NIfTI | Yes | Yes | MRI | No | No | /Patient/MR_StorT2 | dose_interpolated.nii.gz | Interpolated and resampled dose distribution matrix to MRI geometry. |
| Bladder | NIfTI | Yes | Yes | MRI and sCT | Yes | Yes | /Patient/MR_StorT2 /Patient/sCT | mask_Bladder.nii.gz | Bladder segmentation. Originally created from Spectronic MriPlanner and manually adjusted when required. |
| BODY | NIfTI | Yes | Yes | MRI and sCT | No | Yes | /Patient/MR_StorT2 /Patient/sCT | mask_BODY.nii.gz | Body contour segmentation. Automatically created in the treatment planning system Eclipse. |
| CTVT_427 | NIfTI | Yes | Yes | MRI and sCT | No | Yes | /Patient/MR_StorT2 /Patient/sCT | mask_CTVT_427.nii.gz | Clinical prostate target volume (CTV), prescribed total dose of 42.7 Gy, manually segmented by an oncologist. |
| CTVT_427_nnUnet_fold_n | NIfTI | No | Yes | MRI | Yes | No | /Patient/MR_StorT2/nnUNetOutput/folds | mask_CTVT_427_nnUNet_fold_n.nii.gz | nnUNet prostate CTV segmentation from fold n (n=0:9). |
| CTVT_427_nnUnet | NIfTI | No | Yes | MRI | Yes | No | /Patient/MR_StorT2/nnUNetOutput | mask_CTVT_427_nnUNet.nii.gz | Final nnUNet prostate CTV segmentation. |
| CTVT_427_nnUNet_uncertaintyMap | NIfTI | No | Yes | MRI | Yes | No | /Patient/MR_StorT2/nnUNetOutput | mask_CTVT_427_nnUNet_uncertaintyMap.nii.gz | nnUNet prostate CTV segmentation uncertainty map. |
| CTVT_427_step1_obsN | NIfTI | No | Yes | MRI | No | No | /Patient/MR_StorT2/observerData | mask_CTVT_427_step1_obsN.nii.gz | nnUNet prostate CTVT_427 segmentation after editing without uncertainty map in step1, obsB-obsE. |
| CTVT_427_step2_obsN | NIfTI | No | Yes | MRI | No | No | /Patient/MR_StorT2/observerData | mask_CTVT_427_step2_obsN.nii.gz | nnUNet prostate CTVT_427 segmentation after editing with uncertainty map in step2, obsB-obsE. |
| FemoralHead_R | NIfTI | Yes | Yes | MRI and sCT | Yes | Yes | /Patient/MR_StorT2 /Patient/sCT | mask_FemoralHead_R.nii.gz | Femoral head segmentation, patient right side. |
| FemoralHead_L | NIfTI | Yes | Yes | MRI and sCT | Yes | Yes | /Patient/MR_StorT2 /Patient/sCT | mask_FemoralHead_L.nii.gz | Femoral head segmentation, patient left side. |
| Genitalia | NIfTI | Yes | Yes | MRI and sCT | No | Yes | /Patient/MR_StorT2 /Patient/sCT | mask_Genitalia.nii.gz | Genitalia segmentation. |
| PenileBulb | NIfTI | Yes | Yes | MRI and sCT | No | Yes | /Patient/MR_StorT2 /Patient/sCT | mask_PenileBulb.nii.gz | Penile bulb segmentation. |
| PTVT_427 | NIfTI | Yes | Yes | MRI and sCT | No | Yes | /Patient/MR_StorT2 /Patient/sCT | mask_PTVT_427.nii.gz | Planning target volume (PTV). This is the prostate CTV with added 7 mm isotropic margin to account for error sources in radiotherapy treatment. |
| Rectum | NIfTI | Yes | Yes | MRI and sCT | No | Yes | /Patient/MR_StorT2 /Patient/sCT | mask_Rectum.nii.gz | Rectum segmentation. |
| Rectum_nnUnet_fold_n | NIfTI | No | Yes | MRI | Yes | No | /Patient/MR_StorT2/nnUNetOutput/folds | mask_Rectum_nnUNet_fold_n.nii.gz | nnUNet rectum segmentation from fold n (n=0:9). |
| Rectum_nnUNet | NIfTI | No | Yes | MRI | Yes | No | /Patient/MR_StorT2/nnUNetOutput | mask_Rectum_nnUNet.nii.gz | Final nnUNet rectum segmentation. |
| Rectum_nnUNet_uncertaintyMap | NIfTI | No | Yes | MRI | Yes | No | /Patient/MR_StorT2/nnUNetOutput | mask_Rectum_nnUNet_uncertaintyMap.nii.gz | nnUNet rectum segmentation uncertainty map. |
| Rectum_step1_obsN | NIfTI | No | Yes | MRI | No | No | /Patient/MR_StorT2/observerData | mask_Rectum_step1_obsN.nii.gz | nnUNet rectum segmentation after editing without uncertainty map in step1, obsB-obsE |
| Rectum_step2_obsN | NIfTI | No | Yes | MRI | No | No | /Patient/MR_StorT2/observerData | mask_Rectum_step2_obsN.nii.gz | nnUNet rectum segmentation after editing with uncertainty map in step2, obsB-obsE |
